# A century of Illinois hover flies (Diptera: Syrphidae): museum and citizen science data reveal recent range expansions, contractions, and species of potential conservation significance

**DOI:** 10.1093/jisesa/iead051

**Published:** 2023-08-03

**Authors:** C Scott Clem, Lily V Hart, Thomas C McElrath

**Affiliations:** Department of Entomology, University of Georgia, 120 Cedar Street, 413 Biological Sciences Building, Athens, GA 30602, USA; Department of Entomology, University of Illinois at Urbana–Champaign, 320 Morrill Hall, 505 S. Goodwin Ave., Urbana, IL 61801, USA; Illinois Natural History Survey, Prairie Research Institute, University of Illinois at Urbana–Champaign, 1816 South Oak Street, MC 652, Champaign, IL 61820, USA; Department of Entomology, University of Illinois at Urbana–Champaign, 320 Morrill Hall, 505 S. Goodwin Ave., Urbana, IL 61801, USA; Illinois Natural History Survey, Prairie Research Institute, University of Illinois at Urbana–Champaign, 1816 South Oak Street, MC 652, Champaign, IL 61820, USA

**Keywords:** checklist, faunistics, specimens, biodiversity, pollinators

## Abstract

Hover flies of the family Syrphidae are a highly diverse group of insects that exhibit varied life histories and provide numerous ecosystem services. Despite their importance, they are highly understudied, and many biological and distributional patterns remain unknown in regions like the midwestern United States. Data from specimens exist in regional insect collections but is largely undigitized and thus inaccessible to much of the scientific community. Here, we report our efforts to identify, recurate, and digitize thousands of specimens from the Illinois Natural History Survey Insect Collection. We then combine these data with existing datasets to compile a comprehensive checklist of Illinois hover fly fauna, assess for temporal range expansion/contraction trends, and identify species of potential conservation significance. All total, the over 20,000 specimens/records we examined revealed 209 species within 71 genera and all 4 subfamilies of Syrphidae to have ever occurred in Illinois. Based on previously published data, 68 of these species are new Illinois state records and 36 expand the previously known range significantly. Numerous species found in Illinois historically have only recently been reported further north, while others of historically southern distribution appear to be extending their range northward, possibly due to anthropogenic factors like climate change. Furthermore, 73 species have not been reported in Illinois since at least 1995, and 27 are deemed to be of potential conservation significance with few to no recent records in the Midwest or elsewhere. Our findings illustrate the importance of routine expansion, curation, and digitization of natural history collections.

## Introduction

**Fig. 1.**
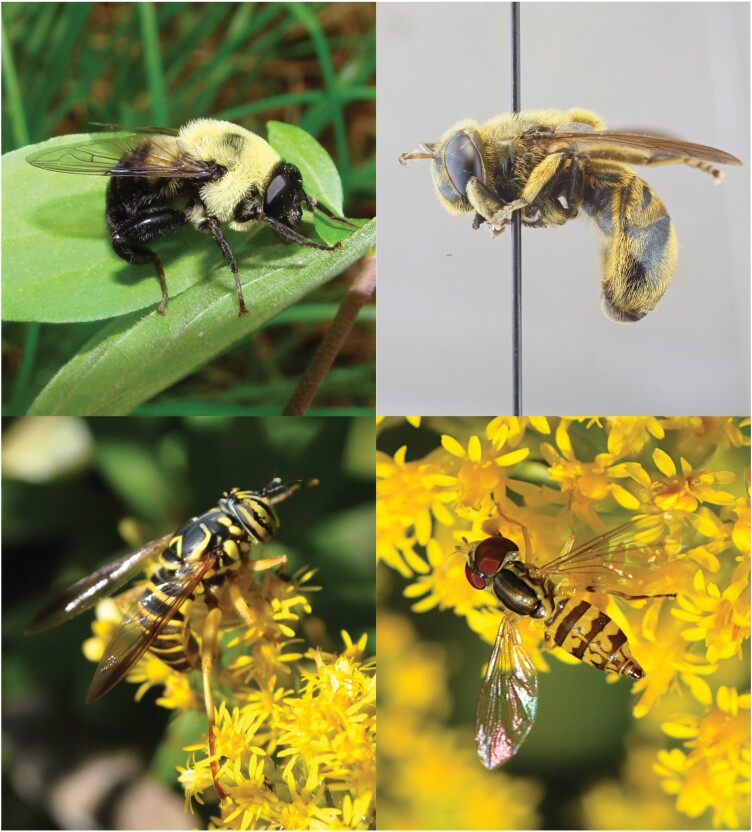
Examples of hover fly digital (photographic) and museum specimen records. Top left: *Mallota posticata*, top right: *Microdon aurulentus*, bottom right: *Toxomerus geminatus*, bottom left: *Spilomyia longicornis*. Photographer credits: Lee Elliott (*M. posticata*) and C. Scott Clem (*M. aurulentus*, *T. geminatus*, and *S. longicornis*).

At approximately 6,200 species within 200 genera worldwide, hover flies (also known as flower flies) of the Diptera family Syrphidae are a highly diverse group of insects that provide a multitude of ecosystem services varying by life cycle and stage (Skevington et al. 2019) (Fig. 1). Adults are significant pollinators for a variety of plant species including many crops (Orford et al. 2015, Rader et al. 2015), amounting to an estimated $300 billion per year in gross global economic value (Doyle et al. 2020). Hover fly larvae occupy multiple niches from reducing environmental contamination via nutrient recycling to biological control of soft-bodied pests such as aphids (Vockeroth 1992, Marshall 2012, Dunn et al. 2020). Many species are also migratory, and their ecological services may be distributed across massive spatial scales (Wotton et al. 2019, Clem et al. 2022). Despite their enormous significance, these insects are highly understudied and many aspects of their diversity, distribution, and conservation remain unresolved.

**Fig. 2.**
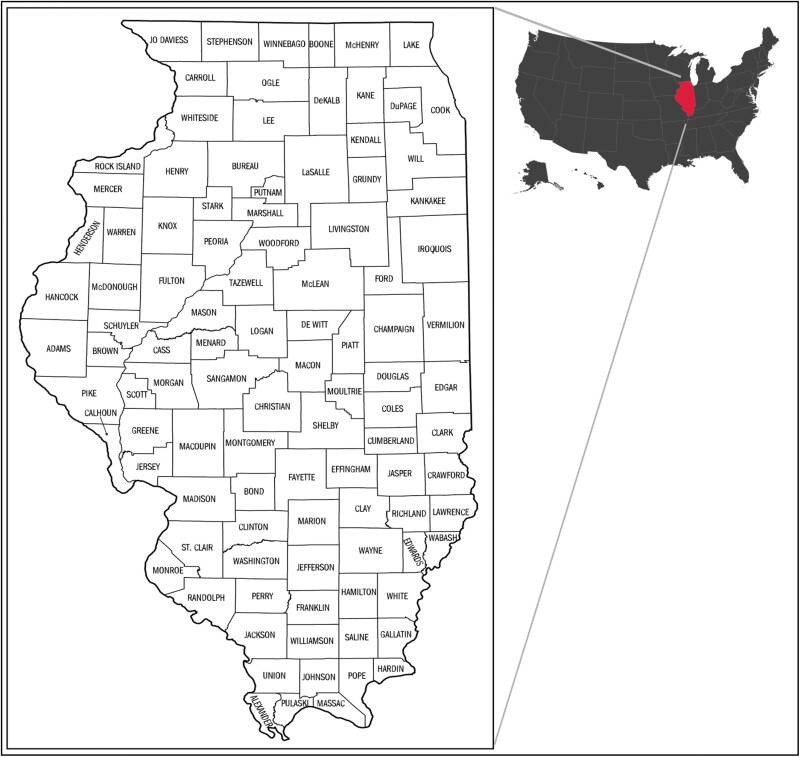
Map of Illinois counties, latitude 36.9540°–42.4951° N, longitude: 87.3840°–91.4244° W.

A total of 828 species of Syrphidae representing all 4 subfamilies (Eristalinae, Microdontinae, Pipizinae, and Syrphinae) are recognized to inhabit North America, with approximately half of these (413) recorded from the northeast (Skevington et al. 2019). Precise knowledge of species distributions is far from complete, especially in states like Illinois. Firmly situated in the midwestern region of the United States (Fig. 2), Illinois occupies an area of 146,942 km^2^, 76% of which is dedicated to agriculture, and 6.5% to urbanization concentrated in the northeastern corner (Luman et al. 2004). Natural ecosystems range from remnant savannah and tallgrass prairie to temperate deciduous forests, encompassing 25 level IV EPA ecoregions (Woods et al. 2006). Extensive efforts to survey the state’s insect fauna have been conducted through the Illinois Natural History Survey (INHS) which houses an arthropod collection of approximately 7 million specimens dating to the late 1800s (McElrath 2022). The Syrphidae in the collection number in the tens of thousands and were last formally curated by dipterists in the 1970s–1990s (see Acknowledgments), which is also when a large portion of specimens was contributed. INHS is also notable in that it houses the collections of Charles Robertson, who made meticulous recordings of plant–pollinator activity in Carlinville, Illinois in the early 1900s (Marlin and LaBerge 2001, Tooker et al. 2006). A large repository of unidentified specimens has accumulated over several decades, and much data has remained undigitized, unpublished, and not readily available for scientific study.

Our primary goal was to identify, update, and digitize the massive holdings of Illinois Syrphidae in the Illinois Natural History Survey Insect Collection. We then combined these data with 3 other datasets, including one based on citizen science, to create a comprehensive checklist of Illinois hover flies complete with all known county and state records. Finally, we compared contemporary and historical data to identify recent distributional patterns, and species of potential conservation significance. A grander goal of this manuscript was to illustrate the importance of routine expansion, curation, and digitization of natural history collections.

## Materials and Methods

All Illinois Syrphidae residing in the INHS Insect Collection pinned material were examined as part of this study. Unidentified syrphid specimens located in the undetermined Syrphidae and undetermined Diptera sections of the collection were sorted and identified to species (or in some cases genus) using the most current, relevant literature (Vockeroth 1986, 1992, Miranda et al. 2013, Young et al. 2016, Skevington et al. 2019). Previously identified specimens were also examined and confirmed, and outdated synonyms were updated where relevant. Taxonomic experts (see Acknowledgments) were consulted when necessary. This process took approximately one year to complete (August 2020–August 2021).

Data from representative INHS specimens of each species from unique Illinois counties and unique dates were digitized using TaxonWorks (TaxonWorks Community 2022) and uploaded into the Global Biodiversity Information Facility (GBIF) repository using Darwin Core Standards (Wieczorek et al. 2012, GBIF.org 2022a). This dataset was then combined with data from 2 recent literature accounts (Skevington et al. 2019, Chisausky et al. 2020) and iNaturalist data collected by citizen scientists and vetted by CSC (GBIF 2022b, 2023a, iNaturalist.org 2022). Finally, the dataset was cross-checked with historical accounts of Charles Robertson (Tooker et al. 2006), and missed records were noted and included. Species distribution maps were generated using Simplemappr (Shorthouse 2010), and overall county-level species richness and estimated collection effort (i.e., record count) maps were generated using R Studio (Version 4.1.1; R Core Team 2021) packages “ggmap” (Kahle and Wickham 2013) and “usmap” (Lorenzo 2022). New state records were determined based on previously published literature (Tooker et al. 2006, Skevington et al. 2019, Chisausky et al. 2020). All datasets were cleaned using Excel, OpenRefine, and formatted using DarwinCore Standards (Wieczorek et al. 2012). The main part of the dataset was also checked using the GBIF Validator tool (GBIF.org 2023b). They are deposited into the publicly accessible Illinois Databank (Clem et al. 2023).

To identify species of potential conservation concern, the overall dataset was spliced according to record date into approximate 30-year intervals (pre-1935, 1935–1965, 1966–1995, 1996–2022) which was then incorporated into the species distribution maps (Fig. 4A–D). This dataset was then examined to identify species recorded historically (prior to 1995) which have not been recorded recently. Available data from GBIF, iNaturalist, and Skevington et al. (2019) were consulted to ascertain whether these species have been recorded recently in surrounding midwestern states (Iowa, Indiana, Wisconsin, Missouri, Kentucky) and whether species are historically rare or vagrant to the region. Species that are difficult to identify from photographs are noted where relevant.

**Fig. 3.**
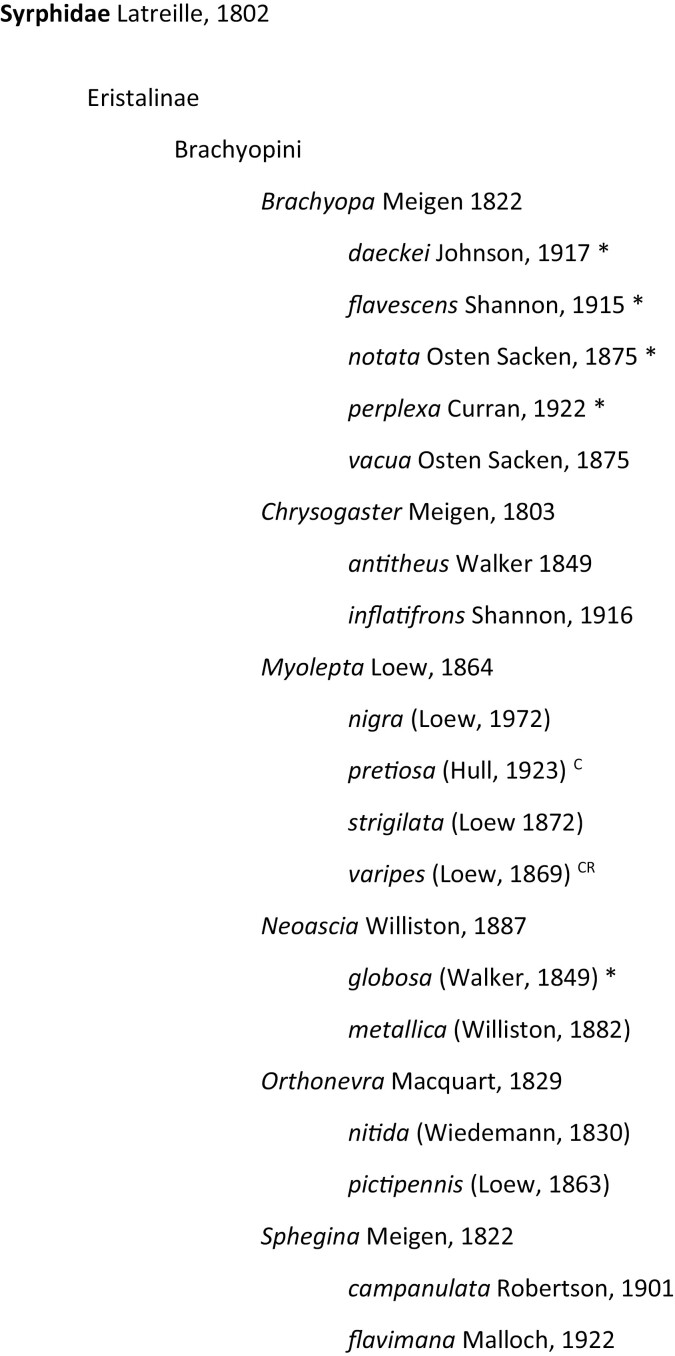

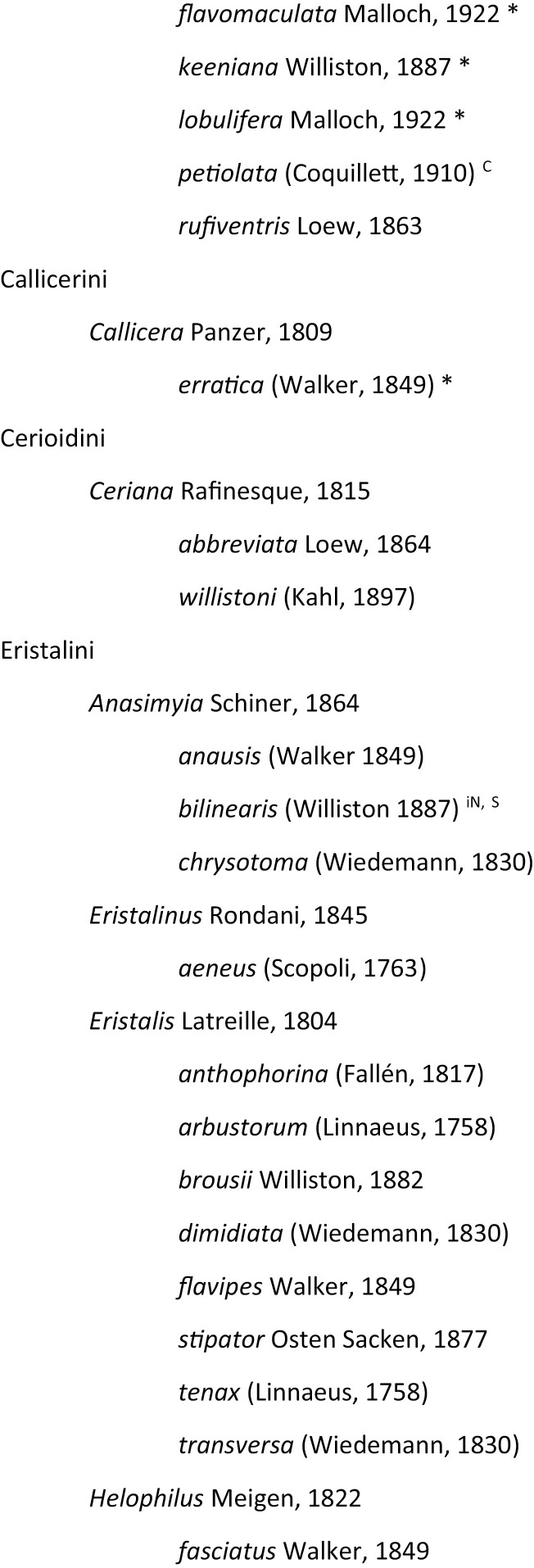

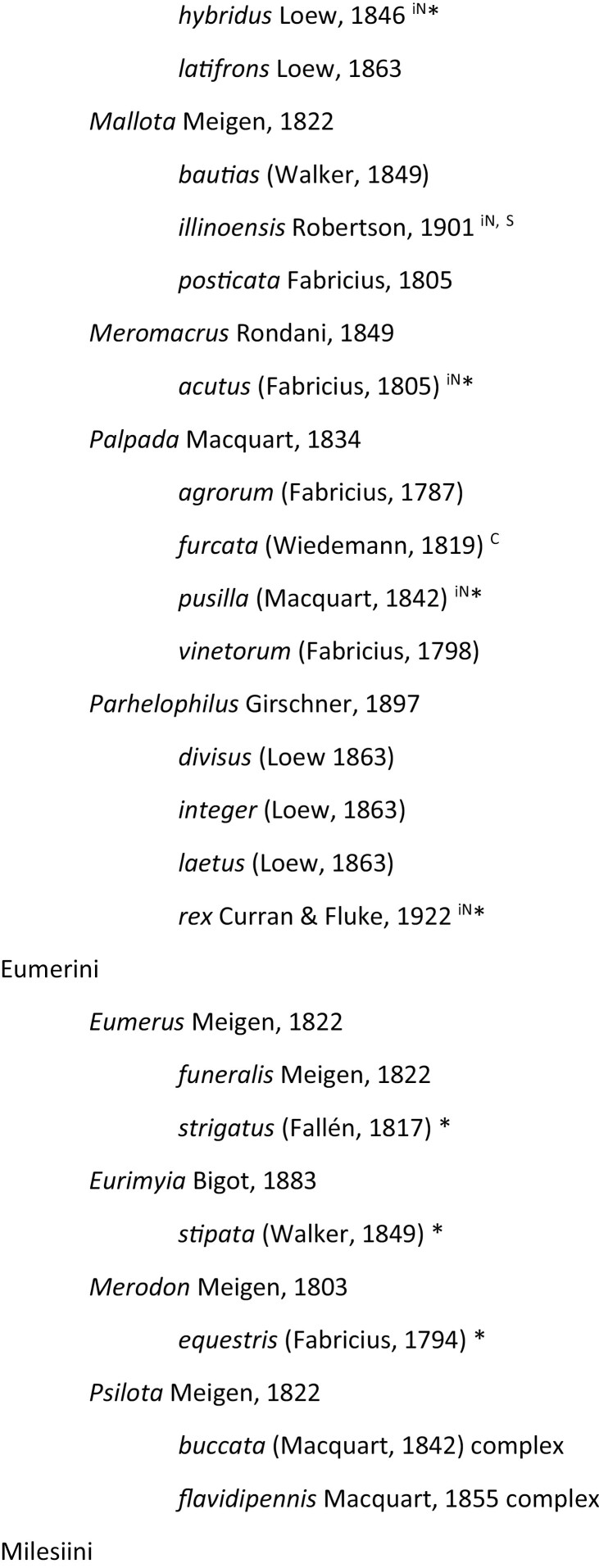

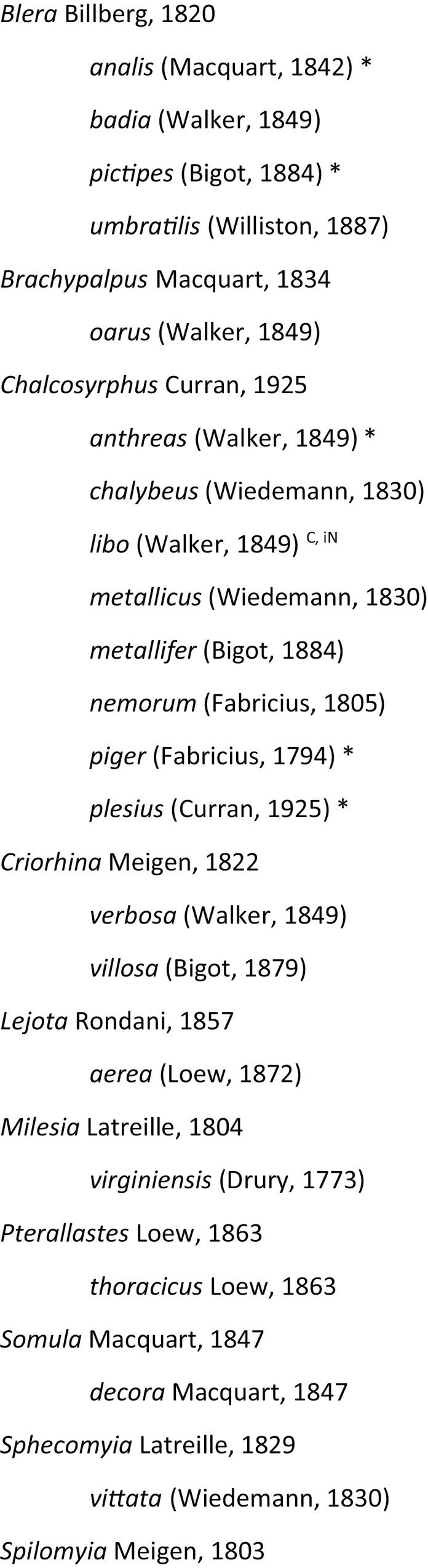

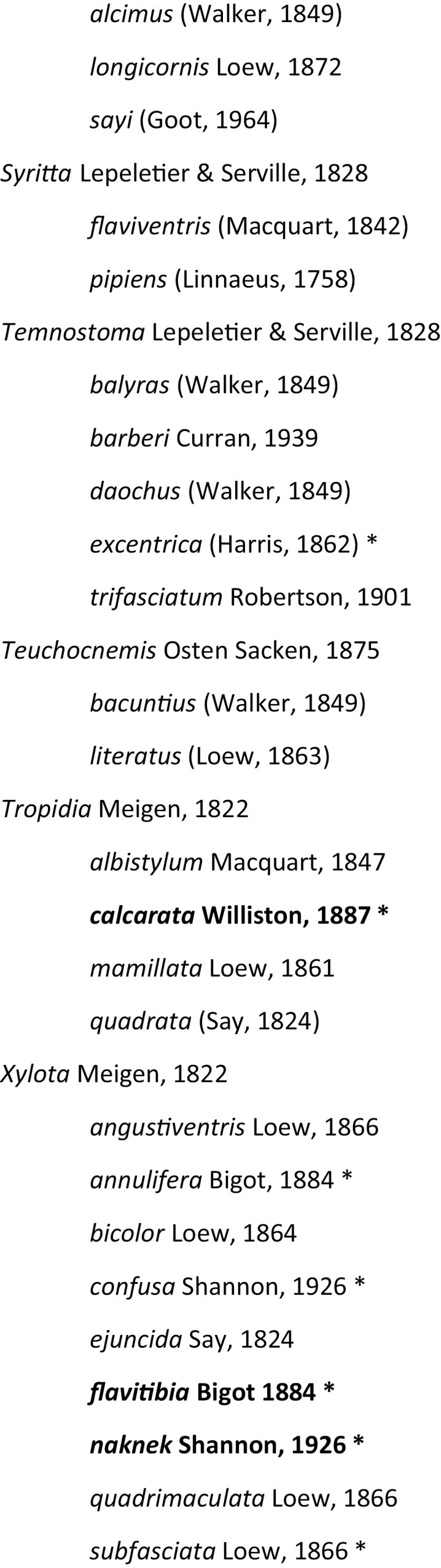

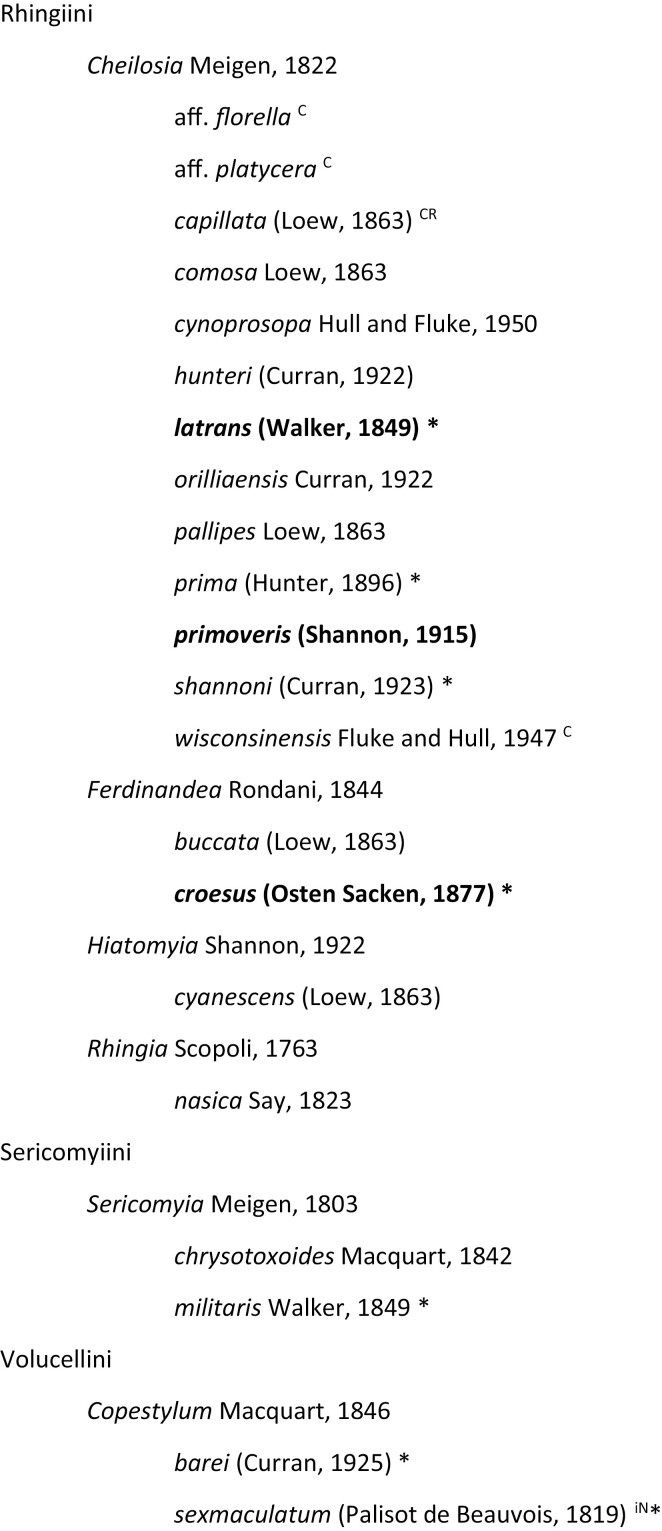

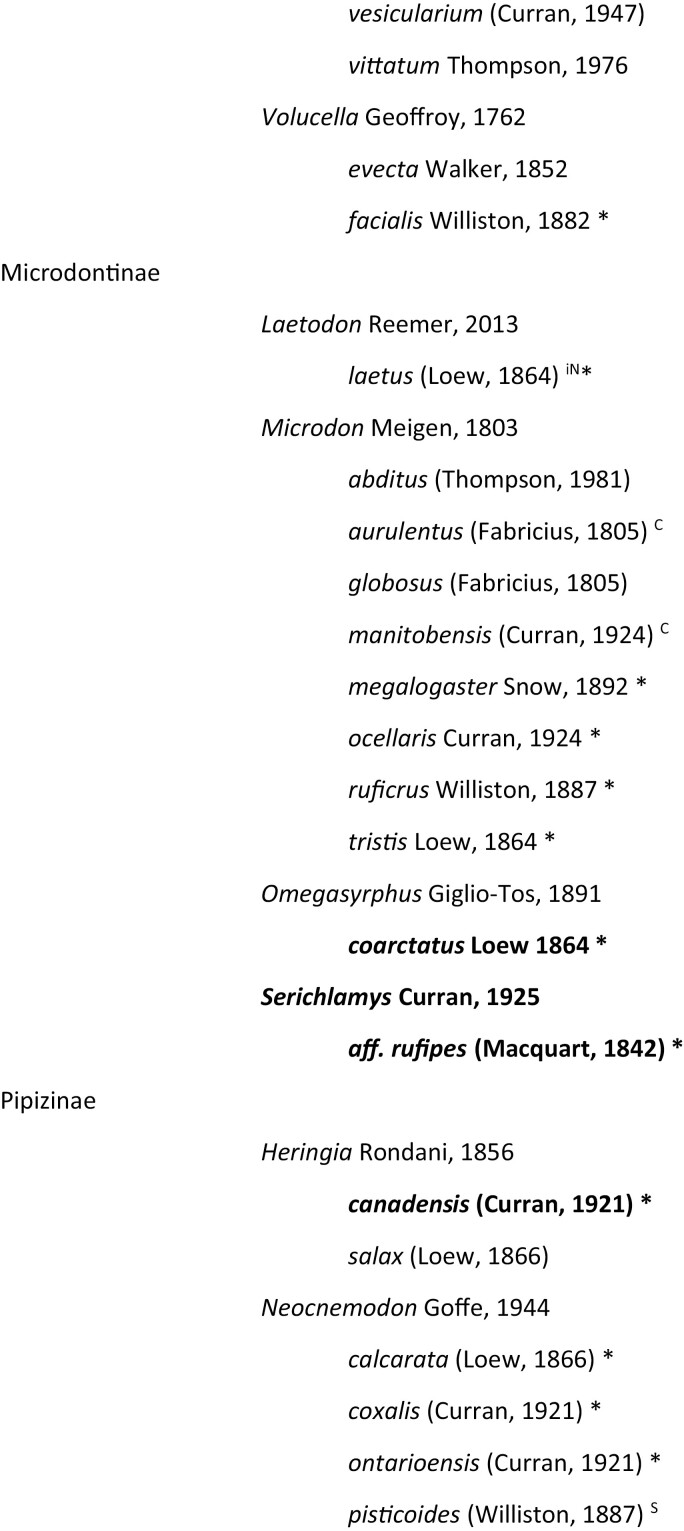

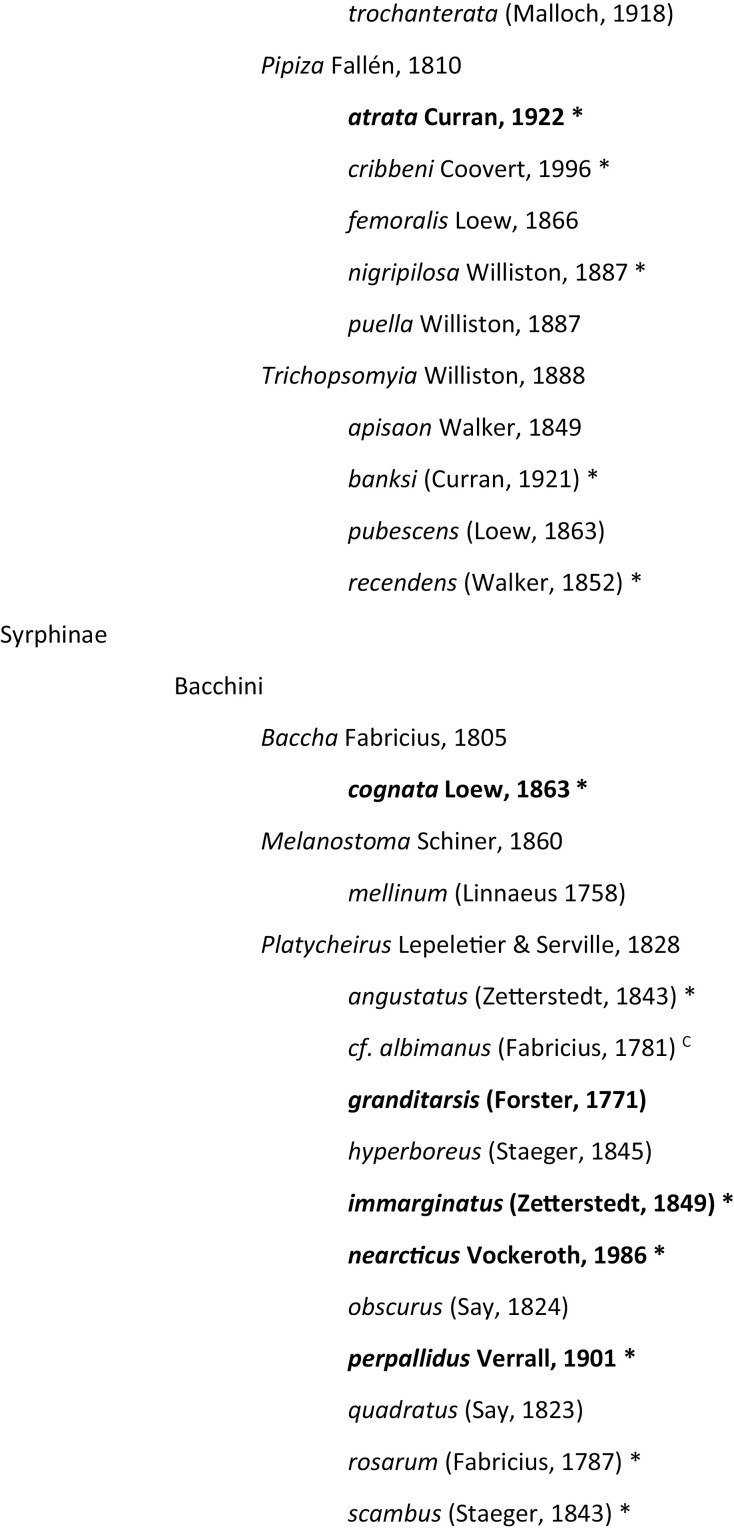

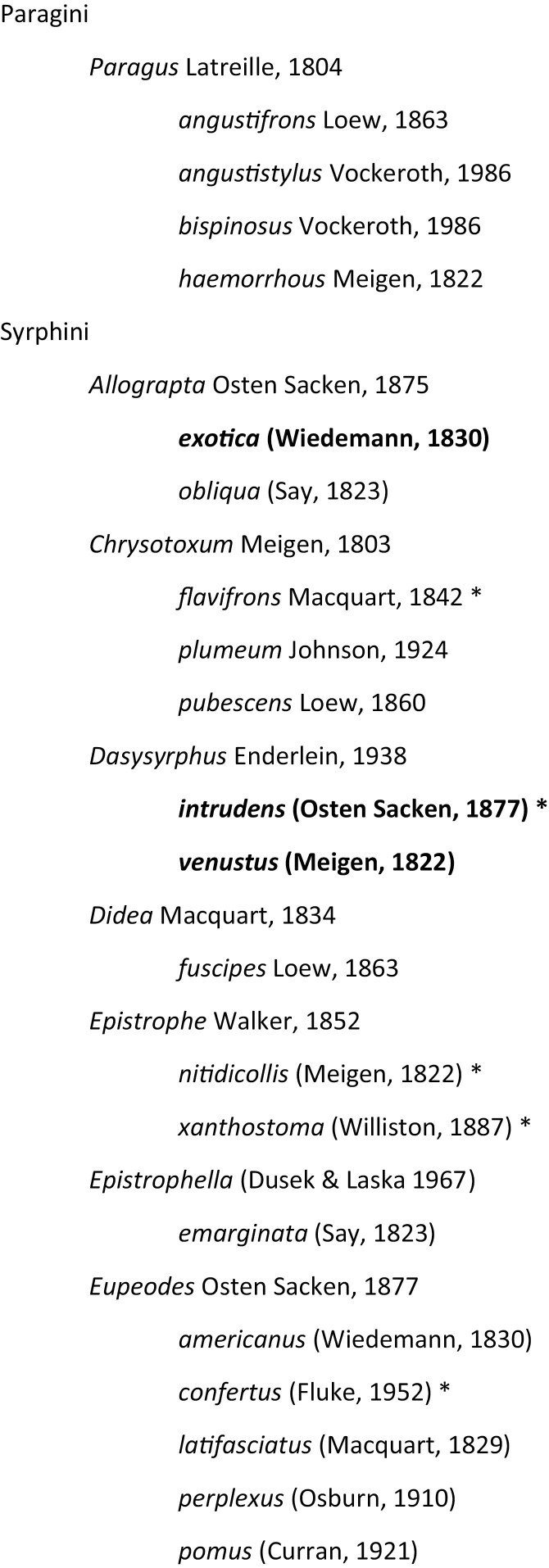

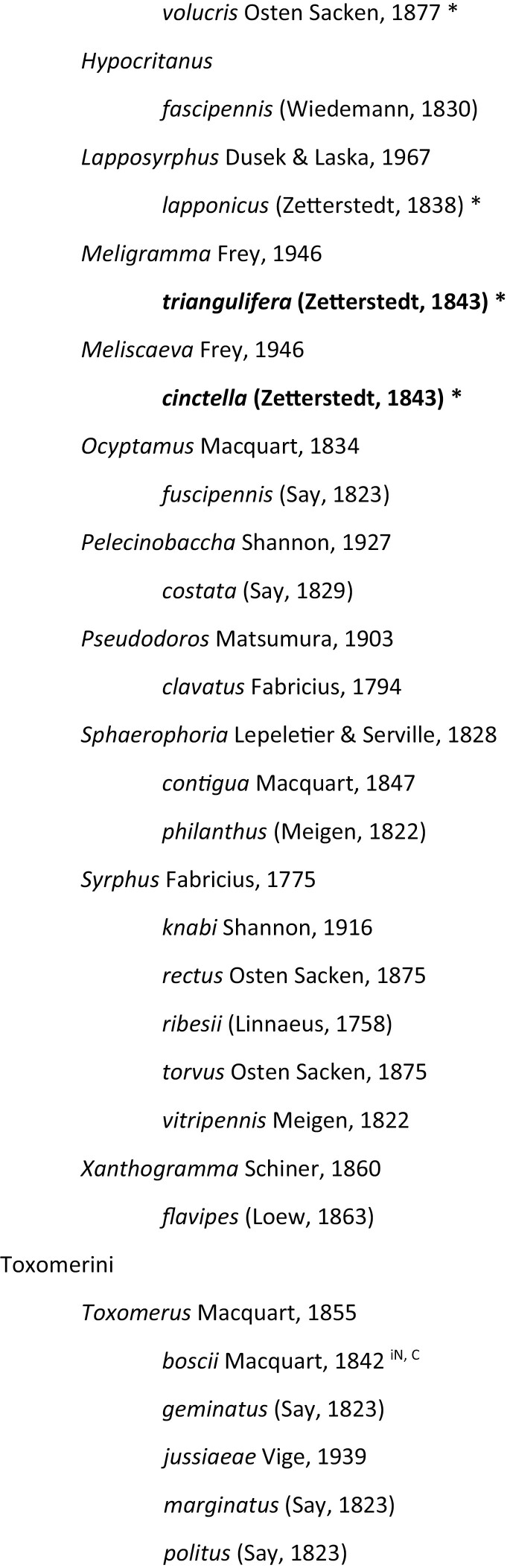
Checklist of Illinois Syrphidae based on INHS Insect Collection holdings and literature records. Bold type signifies new species added to collection, * = new species state records, S = only recorded in Skevington et al. (2019), C = only recorded in Chisausky et al. (2020), iN = only recorded on iNaturalist, CR = recorded by Charles Robertson in Carlinville, IL in 1880s–1890s (Tooker et al. 2006), but no voucher specimens were found.

## Results

**Fig. 4.**
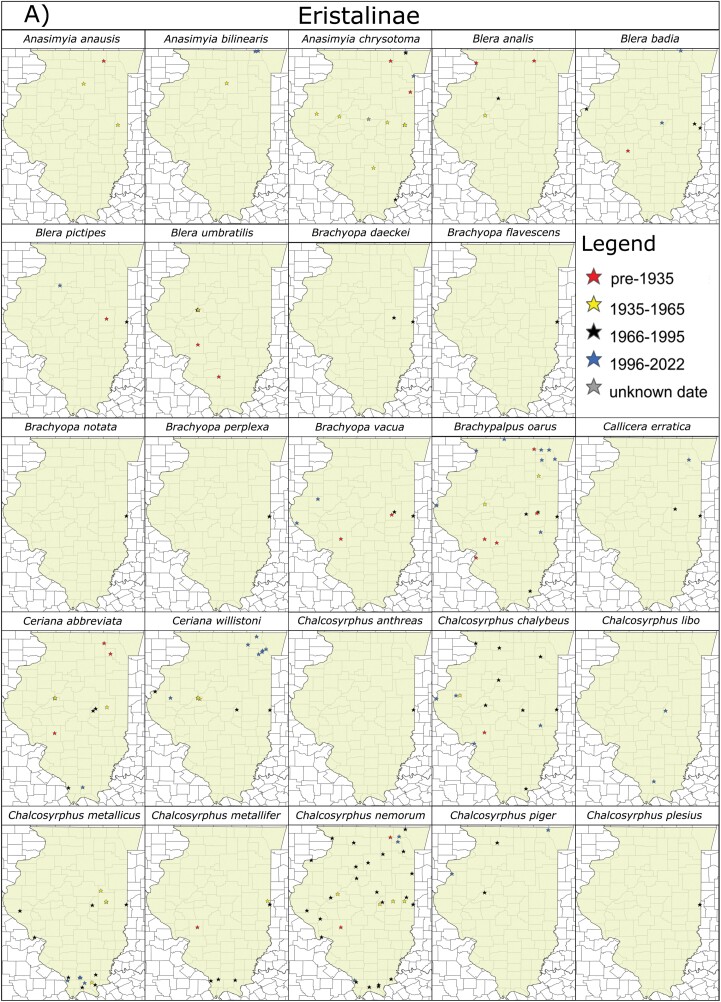

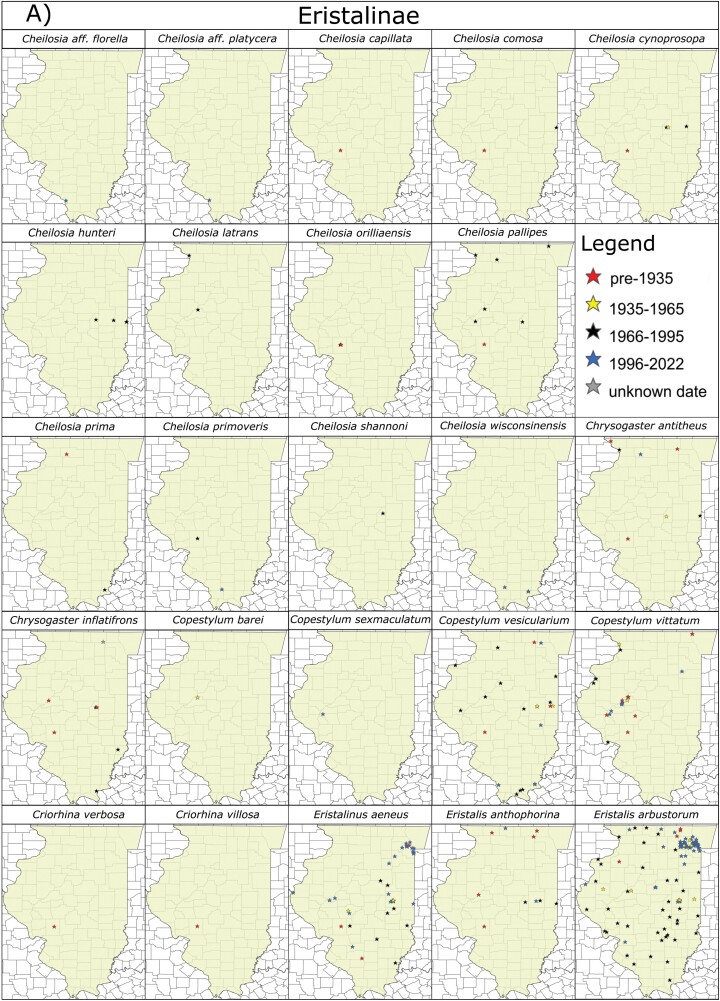

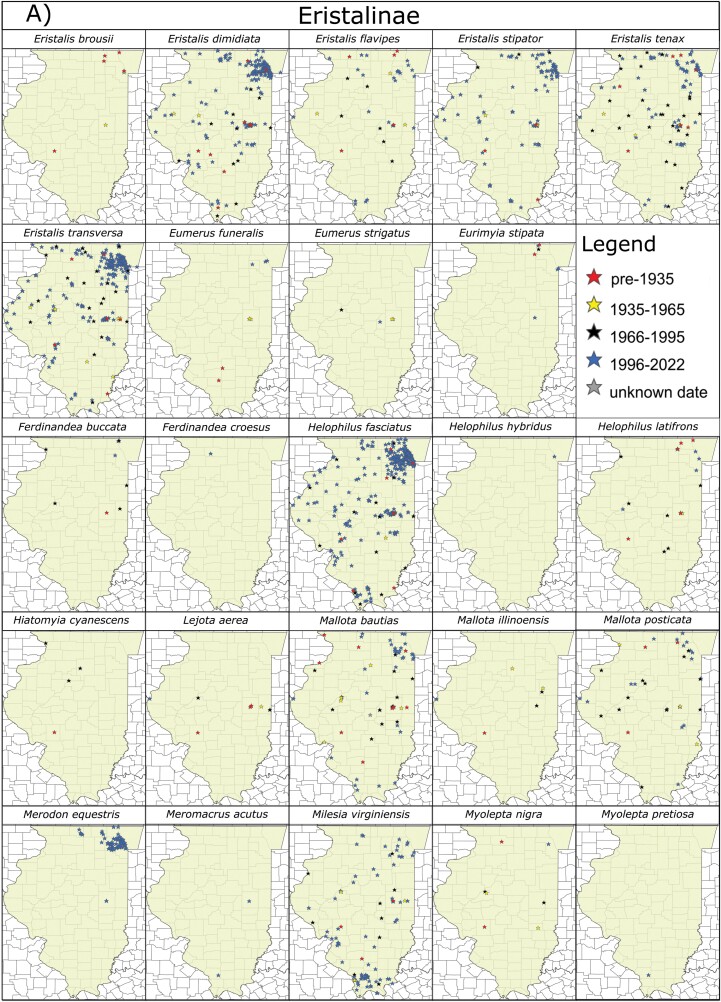

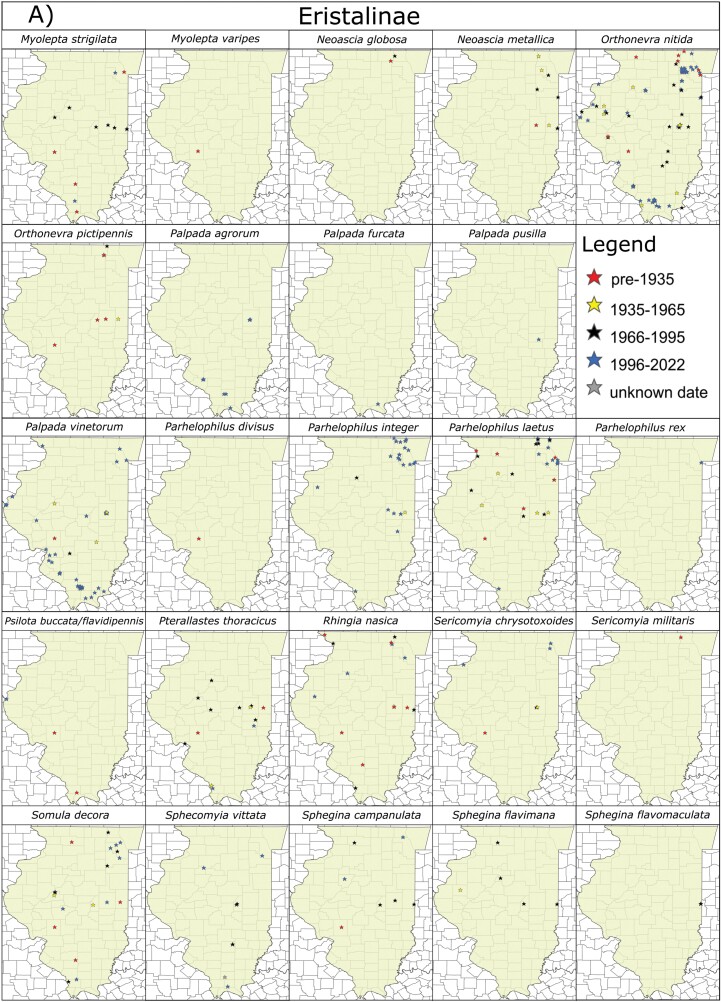

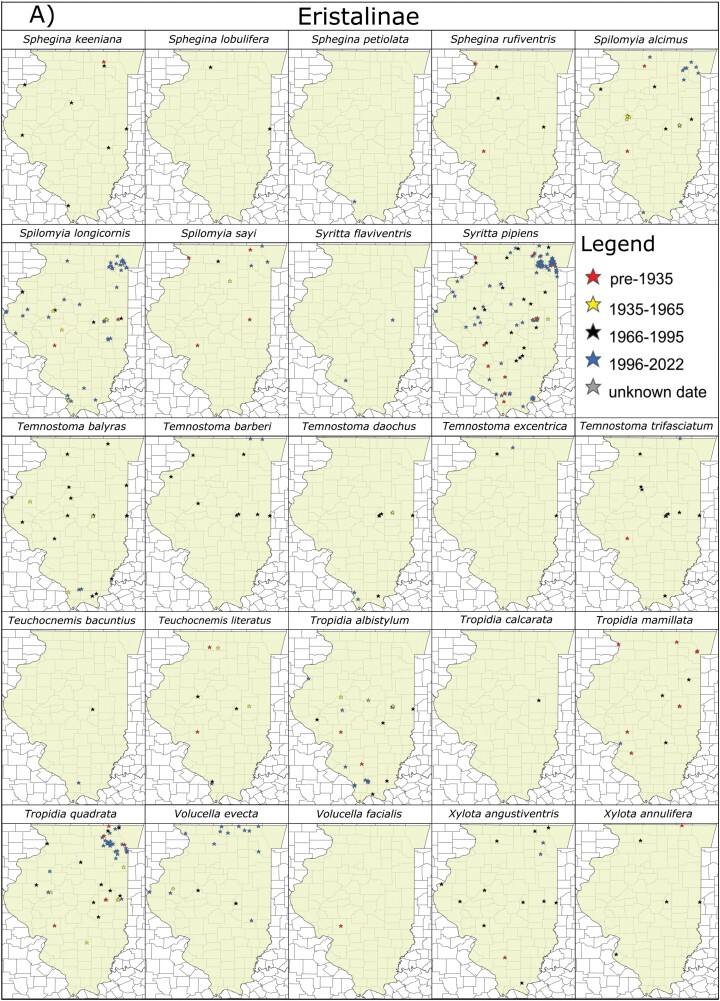

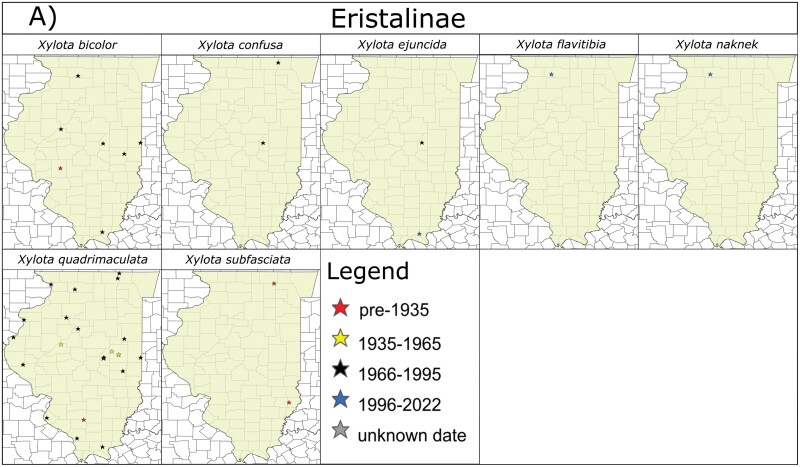

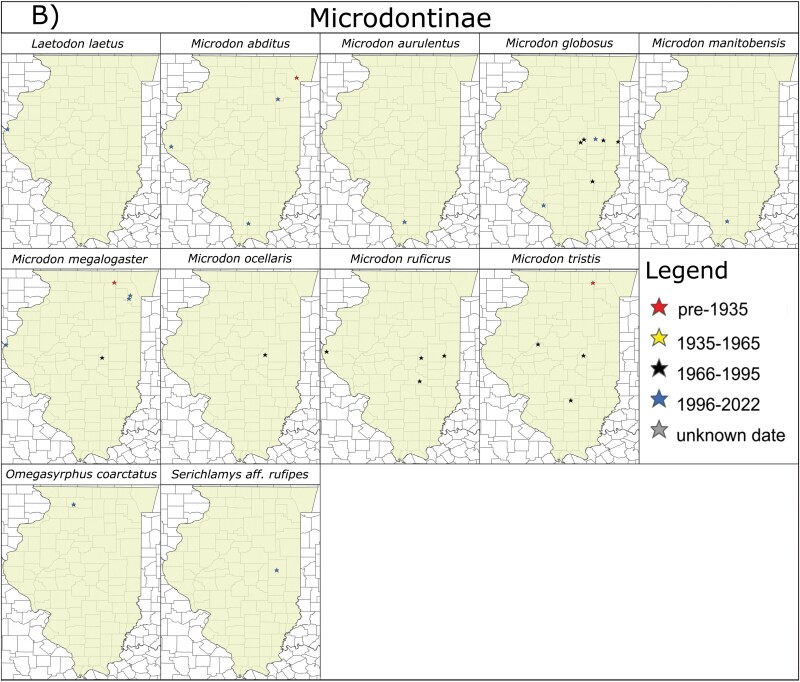

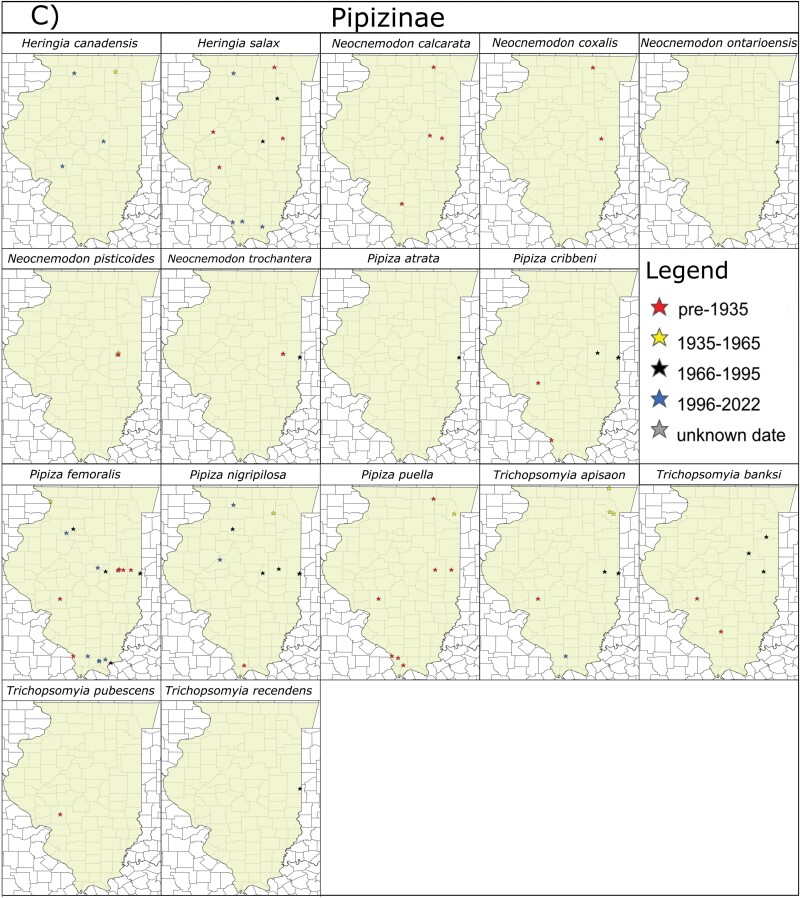

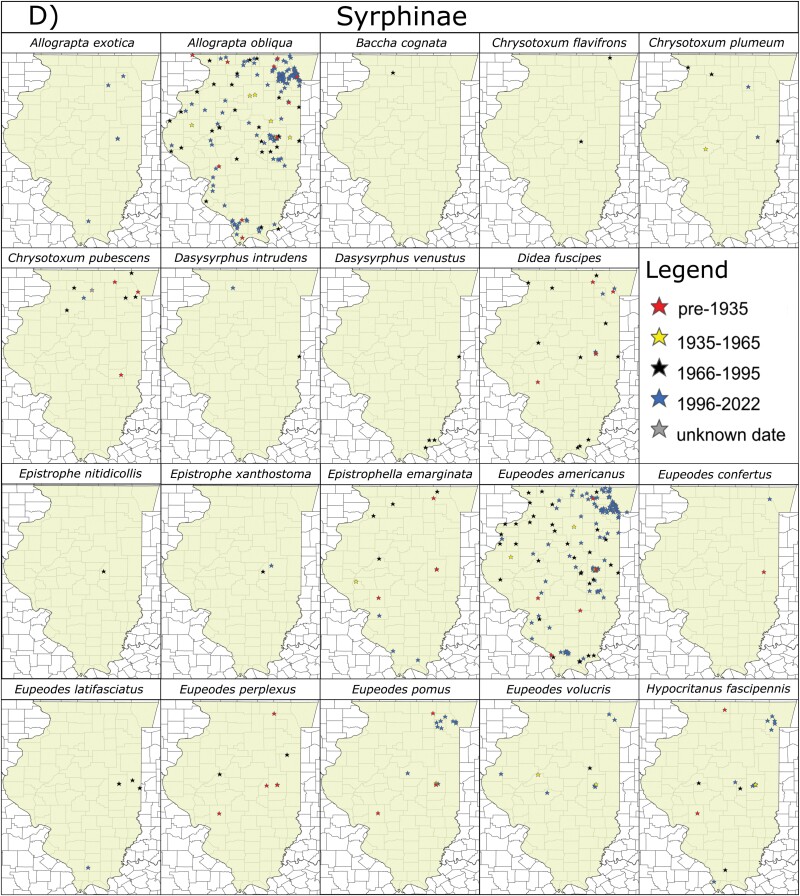

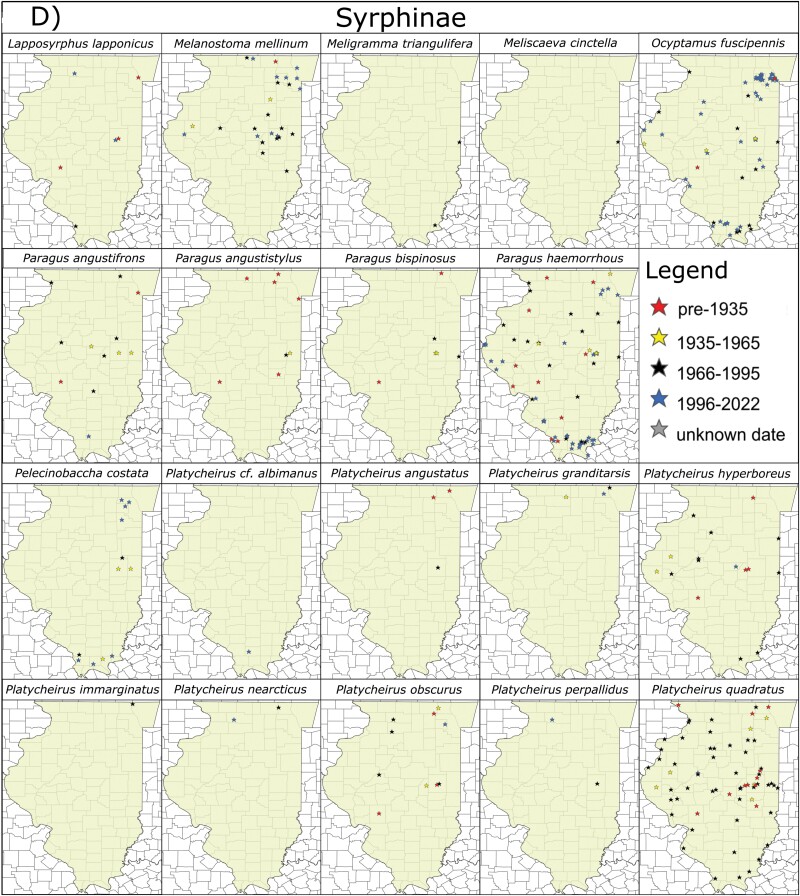

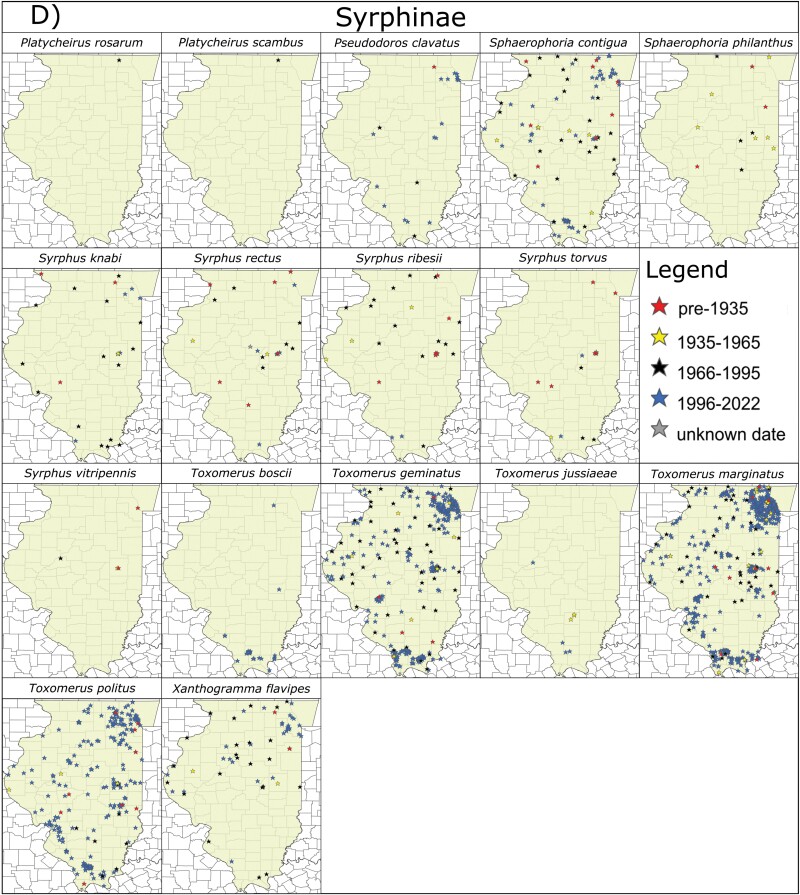
Maps of hover fly (Syrphidae) species distributions within Illinois, organized by subfamily: A) = Eristalinae, B) = Microdontinae, C) = Pipizinae, D) = Syrphinae. Stars represent individual species records compiled from the INHS insect collection, iNaturalist.org 2022 (GBIF Occurrence Download https://doi.org/10.15468/dl.esbaxm), Skevington et al. (2019), Chisausky et al. (2020), and Tooker et al. (2006). Species records are color-coded according to collection year in approximate 30-year intervals: pre-1935, 1935–1965, 1966–1995, and 1996–2022. Records labelled as “unknown date” are specimens with insufficient label data, but which are certainly pre-1995 and probably pre-1935.

**Fig. 5.**
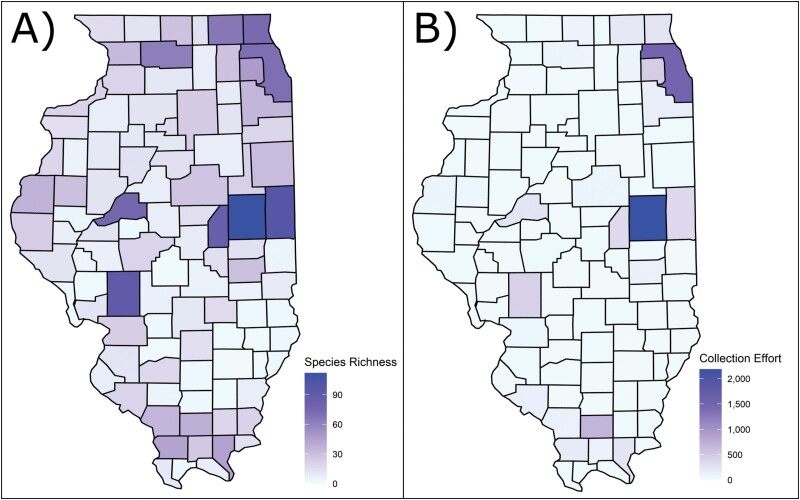
Per county breakdown of sampling effort: A) the number of species collected per county (species richness) and B) the number of specimens recorded per county (collection effort).

Over 20,000 specimens/records were examined as part of this study. Of these, approximately 70% came from the INHS Insect Collection, 21% came from iNaturalist, and 9% came from Skevington et al. (2019) and Chisausky et al. (2020). Over 4,000 previously unidentified syrphid specimens from INHS were identified to species, reducing the number of unidentified Syrphidae by half and adding 20 new species to the collection. In total, 3,900 specimens from INHS were digitized, yielding a combined total dataset with 9,768 records. This dataset revealed 209 species belonging to 71 genera and all 4 subfamilies as having been collected or observed in Illinois (Figs. 3 and 4). We determined 68 of these species to be new Illinois state records, with 36 not recorded from adjacent states, and thus substantially outside of their previously known range according to Skevington et al. (2019). Two species, *Myolepta varipes* (Loew, 1869) and *Cheilosia capillata* (Loew, 1863) were recorded only in Charles Robertson’s historical accounts (Tooker et al. 2006) and could not be verified with voucher specimens. The top 10 Illinois counties with the greatest amount of species records are Champaign (112), Vermillion (99), Macoupin (91), Piatt (85), Mason (75), Lake (73), Cook (70), McHenry (66), and Ogle (62), which are all either in central Illinois and heavily sampled by INHS taxonomists decades ago, or in northeastern Illinois and of high human population density (Fig. 5A and B). Sampling effort has been lowest in southeastern and northwestern counties.

A total of 73 species were identified as having been recorded historically but not recently, and several of these are at the southern or southwestern edge of their historic range (Table 1, see notes). We identified at least 27 species to be of potential conservation significance due to lack of contemporary records in either the Midwest or overall. Finally, at least eight species were identified as being more common in Illinois recently than historically (Table 2, Fig. 4).

**Table 1.** Table of species not recorded in Illinois since at least 1995, with species in bold representing those of potential conservation significance (see Methods)

Species	Number of specimens	Unique records	Most recent record	Notes
ERISTALINAE				
*Anasimyia anausis*	20	3	1962	IL at edge of range, common further north, somewhat difficult to ID from photos
*Blera analis*	8	4	1973	Few Midwest records, most from northeast North America
*Blera umbratilis*	26	4	1975	Generally rare in Midwest, range across eastern North America
*Brachyopa daeckei*	2	2	1977	Few records overall, range across eastern North America
*Brachyopa flavescens*	21	1	1977	Few Midwest records, most from northeast North America
*Brachyopa notata*	2	1	1977	Few Midwest records, most from northeastern and northwestern North America
*Brachyopa perplexa*	9	1	1977	Few Midwest records, most from northeast North America
*Chalcosyrphus anthreas*	6	1	1977	Few Midwest records, most from northeast North America
***Chalcosyrphus metallifer***	**108**	**6**	**1977**	**Few recent records overall**; range across eastern North America, rare species, difficult to ID from photos
*Chalcosyrphus plesius*	2	1	1979	Few Midwest records, most from northeast North America
***Cheilosia capillata***	**0**	**1**	**pre-1935**	**Few recent records overall**; most records from eastern North America, rare species, difficult to ID from photos
***Cheilosia comosa***	**43**	**2**	**1988**	**Few recent records overall**; range across central North America, rare species, difficult to ID from photos
***Cheilosia cynoprosopa***	**23**	**4**	**1982**	**No recent records overall**; rare throughout eastern North America range, difficult to ID from photos
***Cheilosia hunteri***	**57**	**3**	**1982**	**Recent records disproportionate to historical records;** Most records from Canada but few records throughout, difficult to ID from photos
*Cheilosia latrans*	2	2	1984	Few Midwest records, common further north, difficult to ID from photos
*Cheilosia orilliaensis*	3	2	1972	Few Midwest records, wide range but many from northeast North America, difficult to ID from photos
*Cheilosia pallipes*	12	7	1979	Few Midwest records, most from northeast North America, difficult to ID from photos
*Cheilosia prima*	2	2	1989	Few Midwest records, most from northeast North America, difficult to ID from photos
*Cheilosia shannoni*	2	1	1982	Few Midwest records, most from northeast North America, difficult to ID from photos
***Chrysogaster inflatifrons***	**24**	**7**	**1989**	**No recent records overall**; uncommon throughout eastern North America, difficult to ID from photos
*Copestylum barei*	2	1	1961	Few Midwest records, most from southeastern North America, somewhat difficult to ID from photos
***Criorhina verbosa***	**0**	**1**	**1932**	**Range includes Midwest but few recent records**; many recorded from northeast North America, easily identified
***Criorhina villosa***	**2**	**1**	**1894**	**Extremely rare overall**; range throughout eastern North America
***Eristalis brousii***	**32**	**7**	**1939**	**Range includes Midwest but few recent records**; known declining species, somewhat difficult to ID from photos
***Hiatomyia cyanescens***	**34**	**4**	**1986**	**Range includes Midwest but no recent records**; several recorded from northeast North America, somewhat difficult to ID from photos
*Myolepta varipes*	**0**	1	pre-1935	Rare species overall, range across eastern North America
*Neoascia globosa*	2	2	1975	Few Midwest records, most from northeast North America, difficult to ID from photos
*Neoascia metallica*	31	8	1991	Few Midwest records, common further north, difficult to ID from photos
*Orthonevra pictipennis*	57	7	1967	Wide North America range, uncommon, difficult to ID from photos
***Parhelophilus divisus***	**4**	**4**	**1887**	**Range includes Midwest but few recent records**; uncommon species, difficult to ID from photos
*Sericomyia militaris*	2	1	1925	IL at edge of range, common further north
***Sphegina flavimana***	**14**	**5**	**1979**	**Range includes Midwest but few recent records**; many recorded from northeast North America, difficult to ID from photos
*Sphegina flavomaculata*	20	1	1977	Few Midwest records, most from northeast North America, difficult to ID from photos
***Sphegina keeniana***	**16**	**8**	**1992**	**Few recent Midwest records**; most recorded from northeast North America, difficult to ID from photos
*Sphegina lobulifera*	4	2	1979	Few Midwest records and uncommon throughout eastern North America range, difficult to ID from photos
***Sphegina rufiventris***	**14**	**6**	**1984**	**Range includes Midwest and southeast North America but few recent records**; many recorded from northeast North America, difficult to ID from photos
*Tropidia calcarata*	1	1	1980	Rare species overall, most recorded from northeast North America, difficult to ID from photos
***Tropidia mamillata***	**56**	**12**	**2022 (11 historical)**	**Recent records disproportionate to historical records**; most records from Midwest, difficult to ID from photos
*Volucella facialis*	3	1	1900	Few Midwest records, most from northeastern and western North America
*Xylota annulifera*	23	5	1985	Few Midwest records, most from northeastern and northern North America, difficult to ID from photos
***Xylota bicolor***	**10**	**7**	**1992**	**No recent IL records yet several historical**; many recent southeastern and northeastern North America records, easily identified
*Xylota confusa*	2	2	1979	Few Midwest records, most recorded further north, difficult to ID from photos
***Xylota quadrimaculata***	**145**	**23**	**1988**	**No recent IL records yet several historical**; difficult to ID from photos and likely to go undetected
*Xylota subfasciata*	2	2	1914	Few Midwest records, most recorded further north, difficult to ID from photos
MICRODONTINAE				
*Microdon ocellaris*	1	1	1980	Rare species overall, recorded throughout eastern North America, difficult to ID from photos
*Microdon ruficrus*	8	4	1985	Uncommon species overall, recorded throughout eastern North America, difficult to ID from photos
*Microdon tristis*	10	4	1992	Uncommon species overall, recorded throughout North America, difficult to ID from photos
PIPIZINAE				
***Neocnemodon calcarata***	**7**	**4**	**1917**	**No recent Midwest records**; uncommon, most recorded from northeast North America, difficult to ID from photos
***Neocnemodon coxalis***	**4**	**2**	**1914**	**No recent Midwest records**; uncommon, most recorded from northeast North America, difficult to ID from photos
*Neocnemodon ontarioensis*	1	1	1977	Rare species overall, most recorded from northeast North America, difficult to ID from photos
***Neocnemodon pisticoides***	**0**	**3**	**1935**	**No recent records overall**; rare species, range throughout North America, difficult to ID from photos
***Neocnemodon trochanterata***	**17**	**4**	**1977**	**Extremely rare overall**; type specimens from IL are the only known records, difficult to ID from photos
*Pipiza atrata*	4	1	1977	Rare species overall, range throughout North America, difficult to ID from photos
***Pipiza cribbeni***	**98**	**5**	**1977**	**No recent records overall**; most recorded from northeast North America, difficult to ID from photos
***Pipiza puella***	**27**	**8**	**1946**	**No recent Midwest records**; most recorded from northeast North America, difficult to ID from photos
***Trichopsomyia banksi***	**17**	**5**	**1992**	**Few recent records overall**; range throughout eastern North America, difficult to ID from photos
***Trichopsomyia pubescens***	**2**	**1**	**1893**	**Most overall records extremely old**; rare species recorded throughout North America, difficult to ID from photos
*Trichopsomyia recedens*	1	1	1977	Rare species in Midwest, range throughout eastern North America, difficult to ID from photos
**SYRPHINAE**				
*Baccha cognata*	2	1	1979	Few Midwest records, most from northeastern and northwestern North America
*Chrysotoxum flavifrons*	6	2	1979	IL at edge of range, common further north, somewhat difficult to ID from photos
*Dasysyrphus venustus*	7	4	1989	Few IL/Midwest records, cosmopolitan, common further north
*Epistrophe nitidicollis*	1	1	1979	Few Midwest records, cosmopolitan, common further north
*Meliscaeva cinctella*	1	1	1977	Few Midwest records, cosmopolitan, most recorded from northeastern and northwestern North America
*Meligramma triangulifera*	3	2	1989	Few Midwest records, cosmopolitan, most recorded from northeastern and northwestern North America
*Platycheirus angustatus*	5	3	1977	Few IL/Midwest records, cosmopolitan, most recorded north of IL, difficult to ID from photos
*Platycheirus immarginatus*	1	1	1978	Few IL/Midwest records, cosmopolitan, most recorded north of IL, difficult to ID from photos
*Platycheirus rosarum*	1	1	1978	Few IL/Midwest records, cosmopolitan, most recorded north of IL, difficult to ID from photos
*Platycheirus scambus*	3	1	1978	Few IL/Midwest records, cosmopolitan, most recorded north of IL, difficult to ID from photos
***Paragus angustistylus***	**14**	**8**	**1983**	**Range includes Midwest but no recent records**; uncommon, needs genitalia dissection so difficult to ID
***Paragus bispinosus***	**15**	**6**	**1977**	**Range includes Midwest but no recent records**; rare, needs genitalia dissection so difficult to ID
*Eupeodes perplexus*	18	7	1980	Few Midwest records, most from northeastern and northwestern NORTH AMERICA
*Sphaerophoria philanthus*	30	13	1980	Few IL/Midwest records, cosmopolitan, most recorded north of IL, difficult to ID from photos
*Syrphus vitripennis*	4	4	1976	Few IL/Midwest records, cosmopolitan, most recorded north of IL, difficult to ID from photos

**Table 2.** Species with substantially more recent records (post-1995) than historical records (pre-1995) based on our dataset

Species	Records recently	Records historically	Notes
ERISTALINAE			
*Merodon equestris*	107	0	Exotic species, pest of ornamental plants, most specimens from Chicagoland area
*Palpada agrorum*	7	0	Historically southern distribution
*Palpada vinetorum*	102	8	Historically southern distribution
*Parhelophilus integer*	25	2	Historically rare in IL and surrounding states, eastern distribution
*Volucella evecta*	15	3	Historically eastern distribution
SYRPHINAE			
*Allograpta exotica*	7	0	Historically southern distribution, appears to be moving northward
*Pseudodoros clavatus*	20	4	Historically southern distribution
*Toxomerus boscii*	32	0	Historically southern distribution

## Discussion

To our knowledge, only 141 species of Syrphidae were known from the published literature to have ever occurred in Illinois prior to this study (Tooker et al. 2006, Skevington et al. 2019, Chisausky et al. 2020), and after thoroughly examining the contents at INHS and iNaturalist, we have boosted that number by 33%. Approximately half of these species' records are substantially outside of their previously known range, indicating that there are still many large knowledge gaps in the understanding of Nearctic hover fly species distributions.

Comparisons between old and new datasets yield interesting patterns. Some species may be exhibiting range shifts due to anthropogenic impacts like climate change. Numerous species with more northern range distributions were recorded historically in Illinois (their southern limit) but have not been found recently (Table 1 see notes). Additionally, at least five species including *Allograpta exotica* (Wiedemann 1830), *Palpada agrorum* (Fabricius, 1787), *Palpada vinetorum* (Fabricius, 1798), *Pseudodoros clavatus* Fabricius, 1794, and *Toxomerus boscii* Macquart, 1842 exhibit historically southern distributions but are now relatively common in Illinois and other Midwestern states according to iNaturalist data and CSC personal observations (Table 2, Fig. 4A and D). *Parhelophilus integer* (Loew, 1863) and *Volucella evecta* Walker, 1852 may also be more common now than historically, although for unapparent reasons. *Merodon equestris* (Fabricius, 1794) (the *Narcissus* bulb fly) has been reported in high numbers by iNaturalist observers in the Chicagoland area, but there are no historical specimens of this species from Illinois in the INHS collection (Fig. 4A). This species is a non-native, minor pest of *Narcissus*, daffodil, and other ornamental plant bulbs (Cranshaw 2004), so this pattern may indicate a recent colonization.

We found at least 27 species with few to no recent records in the Midwest or overall, which suggests that some species may no longer occupy their historic range and may be in decline. This is certainly true for at least one species, *Eristalis brousii* Williston 1882, which has been extirpated from most of its known range (Skevington et al. 2019). Indeed, most INHS specimens of this species are approximately 100 years old (Table 1, Fig. 4A). Many of the species on this list are from the subfamily Pipizinae, where nearly 70% of the species have only been recorded in Illinois historically. One species, *Neocnemodon trochanterata* (Malloch, 1918), is only known from four records (17+ specimens including a type set) prior to 1980 in central Illinois and nowhere else in the world (although future revision may change this, see Skevington et al. 2019). Pipizinae are somewhat nondescript and difficult to identify, but it is intriguing that so few recent Illinois records exist even at the subfamily level, despite numerous historical accounts. Many Pipizinae have specialized life histories in which larvae feed on various gall-forming aphids and arboreal prey (Skevington et al. 2019). Perhaps this makes them particularly vulnerable to anthropogenic impacts such as deforestation and the displacement of native plants.

While evidence is mixed, reports of insect declines are far from unprecedented. Numerous studies, particularly over the past 10 years, have evidenced and stressed the importance of insect declines occurring throughout the world (Hallmann et al. 2017, Van Klink et al. 2020, Wagner et al. 2021). This has even been demonstrated specifically in hover flies (Hallmann et al. 2021, Barendregt et al. 2022), and in using INHS bee specimen data (Burkle et al. 2013). In the absence of standardized historical field data, presence-only data from museums like INHS are often the only source of historical information for many understudied species. Upon digitization, these data can provide valuable insights into temporal population trends and the conservation status of species (Gotelli et al. 2021) but appropriate interpretations can be considerably difficult due to collection biases and limited specimen data (Davis et al. 2023). Our reports here should therefore be viewed as a baseline for future research, and we must express caution about using them to make official conservation decisions. Sampling efforts by INHS taxonomists were greatest in the 1970s and prior, and recent accounts are largely limited to citizen scientist photography via iNaturalist. Many species from Table 1 are also difficult to identify through photography, and thus may be overlooked. On the other hand, just because a species was reported recently does not mean it is not declining. Clearly, more research is needed to determine which species are of true conservation concern. Future digitization of specimens from other North American museums is likely to yield greater clarity. At the very least, our study reveals a great dearth in knowledge about contemporary range distributions for many North American Syrphidae, largely due to reduced taxonomist workforce.

Our findings highlight the importance of curation and digitization of insect collections. While online citizen science efforts like iNaturalist are extremely valuable, photographic identification is limited. Meanwhile, many collections suffer from inadequate funding that precludes them from gathering, processing, and identifying new material. Insect collections around the world have massive backlogs of specimens that contain important records awaiting curation and digitization. Even at INHS, there is still a vast repository of undigitized Syrphidae from outside the state of Illinois. Numerous contemporary specimen records also go unreported because specimen digitization is too often viewed as an afterthought and not a responsibility, especially in non-museum-based research projects. This can be improved when laboratories are equipped with tools and protocols for quick specimen digitization. Priorities and resources should also support expert-led, standardized field surveys and rapid-digitization techniques and technologies. True understanding of conservation needs for important insect groups such as the Syrphidae is quite difficult and complex, but digitization and examination of specimen records as we have done here is a crucial first step. Researchers and funding agencies should strongly consider faunistic inventories like these, so that biodiversity information from collections can become broadly accessible to the scientific community. Only then can researchers begin to piece together the challenging puzzle of large-scale insect biodiversity patterns.
